# SARS-CoV-2 Viral Replication Persists in the Human Lung for Several Weeks after Symptom Onset

**DOI:** 10.1164/rccm.202308-1438OC

**Published:** 2024-01-16

**Authors:** Michele Tomasicchio, Shameem Jaumdally, Lindsay Wilson, Andrea Kotze, Lynn Semple, Stuart Meier, Anil Pooran, Aliasgar Esmail, Komala Pillay, Riyaadh Roberts, Raymond Kriel, Richard Meldau, Suzette Oelofse, Carley Mandviwala, Jessica Burns, Rolanda Londt, Malika Davids, Charnay van der Merwe, Aqeedah Roomaney, Louié Kühn, Tahlia Perumal, Alex J. Scott, Martin J. Hale, Vicky Baillie, Sana Mahtab, Carolyn Williamson, Rageema Joseph, Alex Sigal, Ivan Joubert, Jenna Piercy, David Thomson, David L. Fredericks, Malcolm G. A. Miller, Marta C. Nunes, Shabir A. Madhi, Keertan Dheda

**Affiliations:** ^1^ Centre for Lung Infection and Immunity, Division of Pulmonology, Department of Medicine, University of Cape Town and UCT Lung Institute, Cape Town, South Africa;; ^2^ South African MRC Centre for the Study of Antimicrobial Resistance,; ^3^ Division of Medical Virology,; ^4^ Institute of Infectious Disease and Molecular Medicine,; ^5^ Division of Anatomical Pathology, Department of Pathology, and; ^6^ Division of Critical Care, Department of Anaesthesia and Perioperative Medicine, University of Cape Town, Cape Town, South Africa;; ^7^ Faculty of Infectious and Tropical Diseases, Department of Immunology and Infection, London School of Hygiene & Tropical Medicine, London, United Kingdom;; ^8^ Division of Anatomical Pathology,; ^9^ South African Medical Research Council, Vaccines and Infectious Diseases Analytics Research Unit, and; ^10^ Department of Science and Technology/National Research Foundation South African Research Chair Initiative in Vaccine Preventable Diseases, Faculty of Health Sciences, University of the Witwatersrand, Johannesburg, South Africa;; ^11^ Africa Health Research Institute, Durban, South Africa; and; ^12^ Centre of Excellence in Respiratory Pathogens, Hospices Civils de Lyon and Centre International de Recherche en Infectiologie, Équipe Santé Publique, Épidémiologie et Écologie Évolutive des Maladies Infectieuses, Inserm U1111, CNRS UMR5308, ENS de Lyon, Université Claude Bernard – Lyon 1, Lyon, France

**Keywords:** COVID-19, SARS-CoV-2, virus replication, mechanically ventilated patients, upper respiratory tract

## Abstract

**Rationale:**

In the upper respiratory tract, replicating (culturable) severe acute respiratory syndrome coronavirus 2 (SARS-CoV-2) is recoverable for ∼4–8 days after symptom onset, but there is a paucity of data about the frequency and duration of replicating virus in the lower respiratory tract (i.e., the human lung).

**Objectives:**

We undertook lung tissue sampling (needle biopsy) shortly after death in 42 mechanically ventilated decedents during the Beta and Delta waves. An independent group of 18 ambulatory patients served as a control group.

**Methods:**

Lung biopsy cores from decedents underwent viral culture, histopathological analysis, electron microscopy, transcriptomic profiling, and immunohistochemistry.

**Measurements and Main Results:**

Thirty-eight percent (16 of 42) of mechanically ventilated decedents had culturable virus in the lung for a median of 15 days (persisting for up to 4 wk) after symptom onset. Lung viral culture positivity was not associated with comorbidities or steroid use. Delta but not Beta variant lung culture positivity was associated with accelerated death and secondary bacterial infection (*P* < 0.05). Nasopharyngeal culture was negative in 23.1% (6 of 26) of decedents despite lung culture positivity. This hitherto undescribed biophenotype of lung-specific persisting viral replication was associated with an enhanced transcriptomic pulmonary proinflammatory response but with concurrent viral culture positivity.

**Conclusions:**

Concurrent rather than sequential active viral replication continues to drive a heightened proinflammatory response in the human lung beyond the second week of illness and was associated with variant-specific increased mortality and morbidity. These findings have potential implications for the design of interventional strategies and clinical management of patients with severe coronavirus disease (COVID-19).

**At a Glance Commentary**

**Scientific Knowledge on the Subject**

The widely accepted view, based predominantly using samples from the upper respiratory tract, is that acute severe SARS-CoV-2 infection is characterised by a viral replicative phase in the first week of symptomatic illness followed by a pro-inflammatory immunopathologic phase peaking in the second and third weeks of illness. However, it remains unclear whether detection of SARS-CoV-2 beyond 2 weeks after symptom onset represents active replication-competent virus or residual genomic and/or antigenic material in the tissue.

**What This Study Adds to the Field**

We have identified a, hitherto, undescribed bio-phenotype of acute severe COVID-19 characterised by persisting viral replication in the lung for up to 4 weeks after symptom onset. There was compartment-specific (nasopharynx versus lung) discordance. The phenotype was associated with variant-specific accelerated death, an exaggerated inflammatory response, and attenuated T-cell immunity in the lung. This challenges the traditional view that viral replication occurs during the first 5 to 10 days of illness, which is followed by an effector or hyperinflammatory phase. This is the first study, to our knowledge, to systematically culture virus from the human lung and map out its related clinical determinants, including the human lung transcriptomic profile in severe COVID-19 disease.

Coronavirus disease (COVID-19), caused by the severe acute respiratory syndrome coronavirus 2 (SARS-CoV-2), has been the foremost killer globally over the past 3 years. Case fatality risk in hospitalized patients, particularly in mechanically ventilated patients, during the Beta and Delta waves was particularly high (∼50–70% [1]). Even with the Omicron-related variants, case fatality risk remains significant in elderly and immunocompromised persons and in several countries, including the United Kingdom, Italy, France, Brazil, and prominently in China, where there is now an ongoing epidemic of severe COVID-19 (2–10). Better therapeutic interventions are needed. However, despite considerable research, the pathogenesis of severe COVID-19, relative to viral kinetics, remains incompletely understood.

SARS-CoV-2 detection (ascertained through PCR positivity or antigen detection) can persist for several weeks from symptom onset (11). Postmortem studies have shown persistence of SARS-CoV-2 in tissues detected by PCR and immunohistochemistry for up to several weeks after symptom onset (12, 13). However, the detection of SARS-CoV-2 in these studies may represent not replication-competent virus (detectable only by viral culture) but residual genomic or antigenic material in the tissues. Shedding of replicating virus confirmed through serial viral culture (i.e., *in vitro* replication in human cell lines) from the upper respiratory tract (URT) has been shown to persist for only ∼2–8 days after symptom onset (11, 14–23). These findings have been confirmed in human lung challenge studies with viable pathogen, in which virus was cultured from the URT until a median of 4 days (and a maximum of 10 d) after symptom onset (24). However, hardly anything is known about the compartment-specific duration of actively replicating virus in the lower respiratory tract (LRT), particularly in acute severely ill hospitalized patients receiving mechanical ventilation. We hypothesized that there is compartment-specific uncoupling of viral replication in severe COVID-19; that is, replicating virus can persist in the LRT beyond 10 days after symptoms onset, independent of its persistence in the URT, and this persistence may be associated with an altered pulmonary immunity.

Some of the results of these studies have been previously reported in preprint form (https://www.medrxiv.org/content/10.1101/2023.03.06.23286834v2).

## Methods

### Patients

**Figure 1.**
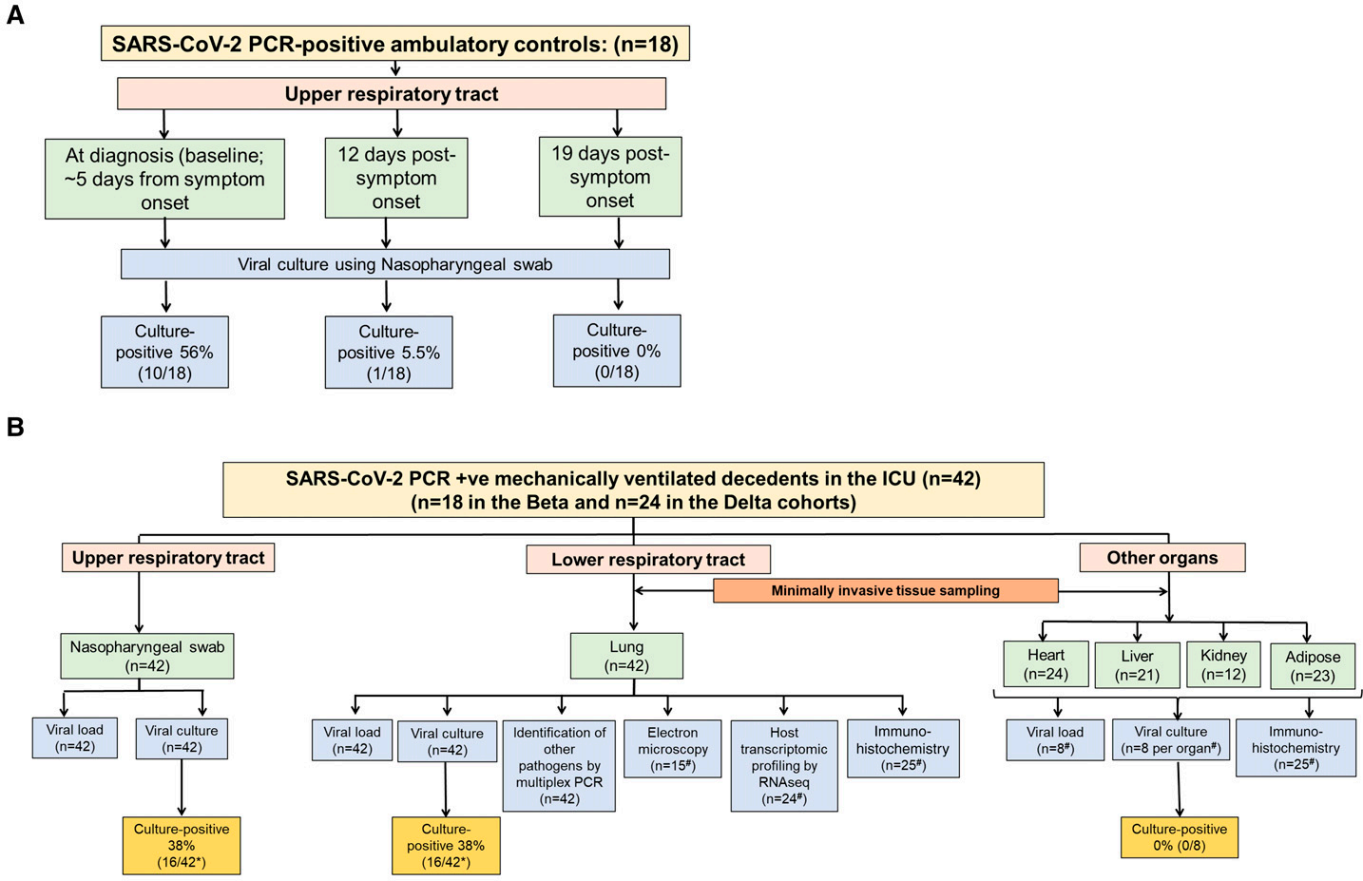
(*A* and *B*) Study overview including SARS-CoV-2 PCR-positive ambulatory control subjects (*A*) and mechanically ventilated decedents (*B*) recruited during the Beta and Delta waves. Nasopharyngeal (NP) swabs from ambulatory control subjects with COVID-19 were obtained approximately 5 days after symptom onset (diagnosis) and then at 12 and 19 days after symptom onset. Minimally invasive tissue samples and NP swabs were retrieved from decedents shortly after death. *The 16 decedents that were nasopharyngeal culture-positive were not the same patients that were lung culture-positive. ^#^Immunohistochemistry, RNAseq, electron microscopy and viral culture of other organs was only performed on the Delta cohort. RNAseq = RNA sequencing; SARS-CoV-2 = severe acute respiratory syndrome coronavirus 2; +ve = positive.

The decedents (*n* = 42) were recruited from Chris Hani Baragwanath Academic Hospital in Johannesburg, South Africa (*n* = 18; Beta group), and Groote Schuur Hospital in Cape Town, South Africa (*n* = 24; Delta group). Figure 1A outlines an overview of the study plan. Ambulatory control subjects (*n* = 18) were recruited at diagnosis (baseline; ∼5 days after symptom onset), 7 and 14 days after diagnosis. Minimally invasive tissue samples and nasopharyngeal swabs from decedents (*n* = 42) in the Beta and Delta waves (Figure 1B) were taken immediately after death. In addition, heart, liver, kidney, and adipose tissue samples were also taken from the Delta variant decedent cohort only. Ethical approval was obtained from the Human Research Ethics Committee (HREC) of the University of Cape Town (HREC approval number 866/2020) and the University of the Witwatersrand (HREC approval number M200313). Biosafety approvals were obtained from the Faculty Biosafety Committee of the University of Cape Town (IBC008-2021).

### Viral Culture

To establish the *in vitro* viral culture model, a SARS-CoV-2 viral stock was used to infect the human lung carcinoma cell line H1299 ACE2 in a Biosafety Level 3 laboratory, and infection was confirmed using light microscopy (as assessed by cytopathic effects of the virus on the cell line) and confocal microscopy (*see* Figures E1A and E1B in the online supplement). Serial dilutions of the viral stock were used to establish the limit of detection of the PCR assay at 1 × 10^1^ copies/ml (*see* Figure E1C). Viral culture was performed on the nasopharyngeal swab and lung biopsy samples as indicated in the study overview (Figure 1) and detailed in the online supplement. Viral culture result reproducibility was good (*see* the online supplement).

### Multiplex PCR to Detect Secondary Bacterial Infections

The lung biopsy cores, stored in universal transport medium, were briefly homogenized, and 200 μl of the supernatant was applied to the BioFire FilmArray Pneumonia panel (Bioméieux). The panel was run using protocol BAL version 3.3 according to the manufacturer’s instructions, thus generating RT-PCR readouts for 33 bacterial and viral pathogens. Bronchopneumonia was defined as histological evidence of a neutrophilic alveolar infiltration together with the detection of bacterial genomic material in the biopsy cores.

### Immunohistochemistry

Immunohistochemical staining was performed using the Ventana automated platform (Ventana XT Autostainer) as indicated by the manufacturer (Roche USA). Tissue sections were prepared, stained, and viewed using standard techniques (25). Antibodies included anti–CD3 (cluster of differentiation 3) (2GV6), and anti-CD8 (SP57) (Roche USA).

### Hematoxylin and Eosin Staining and Transmission Electron Microscopy

Hematoxylin and eosin staining and transmission electron microscopy were performed according to standard procedures (25). Hematoxylin and eosin–stained slides were viewed using a BX43 microscope (Olympus). Transmission electron microscopic tissue sections were viewed using an EM109 microscope (Carl Zeiss).

### SARS-CoV-2 Whole-Genome Sequencing

Total SARS-CoV-2 RNA was extracted from lung biopsy samples and whole-genome sequencing was performed. The generated reads were analyzed using Exatype software (https://exatype.com) to identify minor and major variants. The assembled consensus sequences were analyzed using Nextclade Web (https://clades.nextstrain.org) for further quality control and clade assignment.

### RNA Sequencing

Total RNA was extracted from lung biopsy samples from the Delta group, sequenced, and mapped consecutively to the human and COVID-19 reference genomes using Spliced Transcripts Alignment to a Reference version 2.7.7a (26). A differential expression (DE) analysis was performed on the generated raw read count file using the R package edgeR (version 3.38.4; https://www.ensembl.org) (27). The DE results were ranked by fold change (FC), and the gseGO function, from the R package clusterProfiler (version 4.0) (28), was used to perform a gene set enrichment analysis for Gene Ontology biological process pathways. Pathways with a false discovery rates (FDRs) <0.05 were considered significant.

### Confocal Microscopy

The H1299 ACE2 cells were plated, infected with SARS-CoV-2, and allowed to adhere to coverslip slides overnight at 37°C. The next day, the cells were stained with or without anti–SARS-CoV-2 S1 spike protein (Thermo Fisher Scientific), and the slides were mounted in Mowiol (Calbiochem) containing n-propyl gallate (Sigma-Aldrich) as an antifading agent. Confocal microscopy was performed using an Axiovert 200M LSM510 Meta NLO confocal microscope (Zeiss).

### Sample-Size Calculation and Statistical Analysis

We hypothesized that we would detect lung culture positivity at 14 days after symptom onset in ∼33% of decedents. A sample size of ∼40 participants would allow us to ascertain that degree of positivity with a 15% margin of error using 95% confidence and 80% power (determined using the open-source calculator OpenEpi version 3; https://www.openepi.com).

The Fisher exact test was used for categorical variables, and for continuous variables, the Mann-Whitney test was used for nonparametrically distributed data between the culture-negative and culture-positive groups (Stata version 17 [Quantec] or Prism version 9.4.1 [GraphPad]). A *P* value of <0.05 was considered to indicate statistical significance in all statistical analyses.

Multivariable analysis was performed in R by fitting a binomial generalized linear model to assess the association between steroid use and the presence of secondary bacterial infection on culture status. The R package tidymodels (version 1.0.0; https://tidymodels.tidymodels.org) was used to perform predictive modeling using the glm binomial classification algorithm. To account for the small sample size, 1,000 bootstraps were performed for each analysis using the “bootstraps” function (nonparametric) in the package rsample (version 1.2.0; https://rsample.tidymodels.org).

## Results

### Demographics and Clinical Characteristics of the Decedents

The demographics of patients enrolled in the study are shown in Table E1. The median age of the patients was 53 years, and 48% were men (20 of 42). Secondary bacterial infections were present in 40.5% (17 of 42), and 11% (4 of 38) had bacterial bronchopneumonia (microbiologically and histopathologically confirmed). The median (interquartile range) times from symptom onset to death, ICU admission to death, and high-flow oxygen admission to death were 17 (9–22), 5 (2–12), and 11 (6–15) days, respectively.

### SARS-CoV-2 Replicating Persistence in the Human Lung of Mechanically Ventilated Decedents

**Figure 2.**
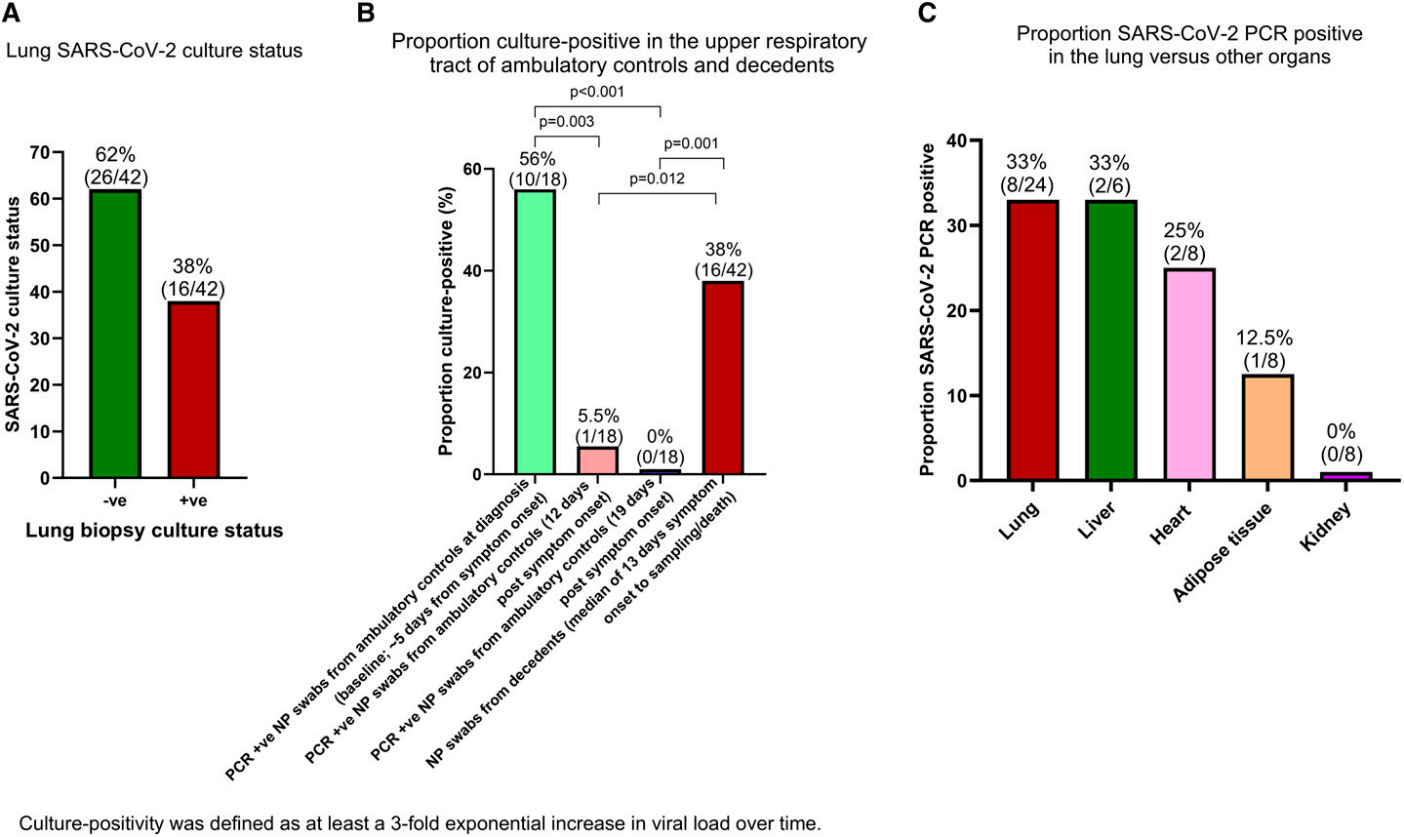
Active replicating virus was recovered from the lungs of more than one-third of decedents (16 of 42). (*A*) Proportion of lung biopsy samples that were culture-positive from the decedents. (*B*) Proportion of ambulatory patient and decedent NP swab samples that were culture-positive. (*C*) PCR positivity of organs of lung culture-positive decedents from the Delta cohort (organs other than the lung were not culture-positive). NP = nasopharyngeal; SARS-CoV-2 = severe acute respiratory syndrome coronavirus 2; -ve = negative; +ve = positive.

**Figure 3.**
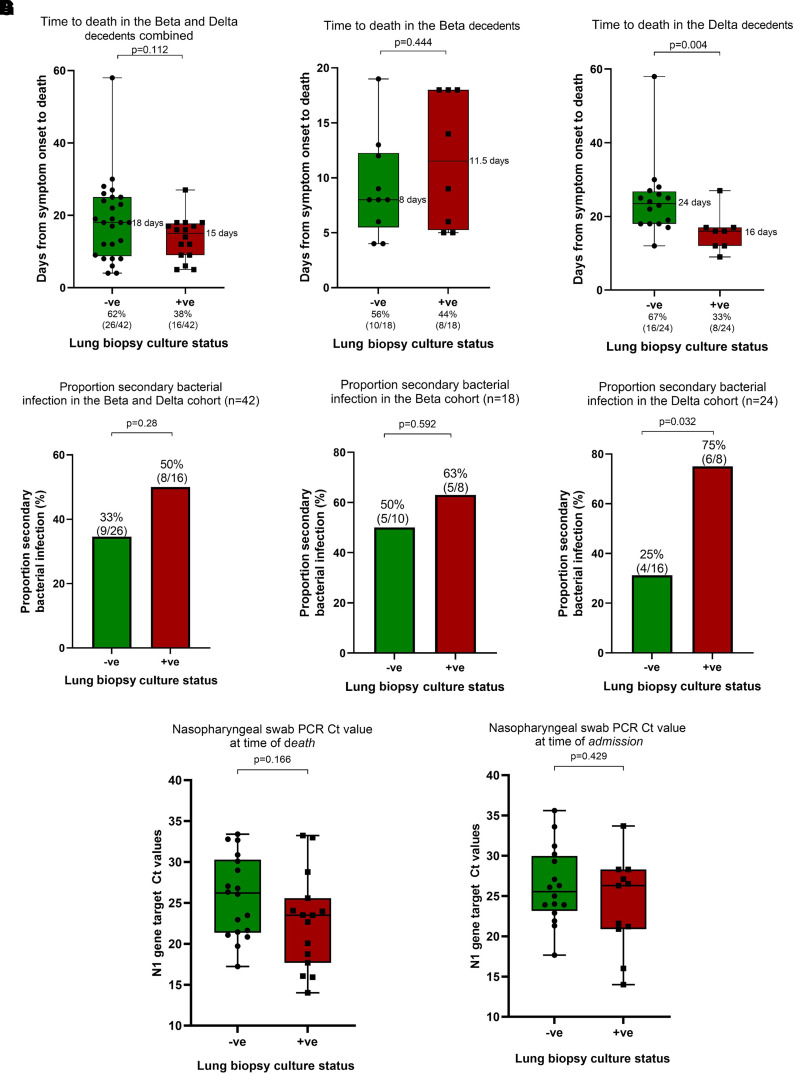
The phenotype of replicating viral persistence, compared with the culture-negative participants, was associated with accelerated death and a higher frequency of bacterial bronchopneumonia in the Delta but not the Beta group. (*A–C*) Days from symptom onset to death for the culture-negative (green) and culture-positive (red) groups for both groups combined (*A*) and for the Beta (*B*) and Delta (*C*) groups individually. (*D–F*) Proportion of samples/participants with secondary bacterial infections in culture-negative and culture-positive decedents overall (i.e., the combined Beta and Delta groups) (*D*), Beta group only (*E*), and Delta group only (*F*). (*G*) PCR cycle threshold (Ct) value at the time of death or at admission could not discriminate or predict lung culture status. The nasopharyngeal swab PCR Ct values at admission or death were missing for some participants because they were either diagnostically confirmed by antigen testing or the Ct value was not recorded. Because of the nature of the pandemic and the burden of the disease on the healthcare infrastructure at the time, Ct values at peak periods were not recorded. We have conducted sensitivity and imputation analyses indicating that these missing data points are redundant. -ve = negative; +ve = positive.

We first ascertained the frequency and duration of replicating virus in lung tissue (which to our knowledge has not been previously undertaken). Culturable virus in the lung was present in 38.1% (16 of 42; Figure 2A) of mechanically ventilated ICU decedents at a median of 15 days (and up to ∼4 weeks; *see* Figure E2) from symptom onset to sampling/death (Figure 3A). As expected, 56% (10 of 18) of a prospectively recruited control group of ambulatory patients had culturable virus, using nasopharyngeal swab samples, at Day 5 after symptom onset (Figure 2B). In the same group of patients at 12 and 19 days after symptom onset, only 5.5% (1 of 18) and 0% (0 of 18), respectively, had culturable virus from their nasopharyngeal swabs (Figure 2B). By contrast, 38% of nasopharyngeal swabs from the mechanically ventilated ICU descendants had culturable virus (Figure 2B) at a median of 13 days from symptom onset to sampling/death (*see* Figure E3B). In addition, SARS-CoV-2 could be detected by PCR in multiple organs in lung culture–positive decedents in the Delta cohort (biopsies other than of the lung were not performed in the Beta cohort), suggesting widespread multiorgan viral dissemination (Figure 2C). SARS-CoV-2 was also detected in adipose tissue of culture-positive decedents (hitherto undescribed). We did not culture virus from the organs of the decedents other than the lung. Thus, viral genetic material was only detectable by PCR in the lung culture–positive patients in the other organs. This probably indicates that the virus disseminated systemically in these patients, who had chronic replicative disease in the lung and not in the other organs. We therefore present only the PCR results from the other organs for the lung culture–positive patient samples in Figure 2C. Clinical characteristics, such as age and comorbidities, were similar in the lung culture–positive and lung culture–negative groups (*see* Table E1). We found no association between viral genetic variant and the phenotype of replicating viral persistence (although this might have been a factor of the limited sample size; *see* Table E5).

### Time to Death in the Delta and Beta Groups and Predictors of Lung Culture Positivity

Next, we evaluated variant-specific relationships to clinical outcomes. Mechanically ventilated patients who were SARS-CoV-2 lung culture positive in the Delta but not the Beta group had a higher proportion of accelerated death (i.e., shorter duration from symptom onset to death; Figure 3C vs. Figure 3B; *P* = 0.004) and a higher proportion of lung-specific secondary bacterial infection (Figure 3F vs. Figure 3E; *P* = 0.032) compared with culture-negative decedents. Similarly to the lung culture data, the nasopharyngeal swab culture–positive Delta group, but not the Beta group, had a higher proportion of accelerated death (*see* Figure E3D vs. Figure E3C; *P* = 0.026).

The bacterial species identified from the lung biopsies of both the Beta and Delta groups included *Streptococcus*, *Staphylococcus*, *Haemophilus*, *Acinetobacter*, *Proteus* spp., *Escherichia*, *Klebsiella*, *Enterobacter*, and *Serratia* (*see* Table E4). Overall, both groups were infected with one or more bacteria that were sensitive or resistant to β-lactams and/or carbapenems (*see* Table E4). Key clinical and demographic characteristics such as differences in comorbidities (age, obesity, diabetes, HIV positivity, etc.; *see* Table E1) associated with drivers of severe COVID-19 and poor prognosis could not explain these observations, despite the lower population-level vaccination and preexisting COVID-19 exposure rates in the Beta cohort. Steroid use (proportion) was similar in the culture-positive and culture-negative groups (though the duration of steroid use was significantly higher in the culture-negative group; *see* Table E1), and there was no significant (*P* > 0.05) association between steroid use and lung culture positivity or the presence of secondary bacterial infection in a multivariable analysis. If anything, there was a trend (*P* = 0.06) toward greater steroid exposure in the lung culture–negative group (in the multivariable analysis), arguing against its role in driving viral replication.

Next, we interrogated whether nasopharyngeal PCR characteristics (cycle threshold [Ct] value), either at admission or close to death, could identify the phenotype of lung replicating viral persistence. However, nasopharyngeal Ct at admission and at the time of death was not associated with lung culture positivity (Figure 3G). This suggests that the kinetics of viral replication were different in the URT and the LRT.

### Lung Immunity and Histology of the Culture-Negative versus Culture-Positive Groups

**Figure 4.**
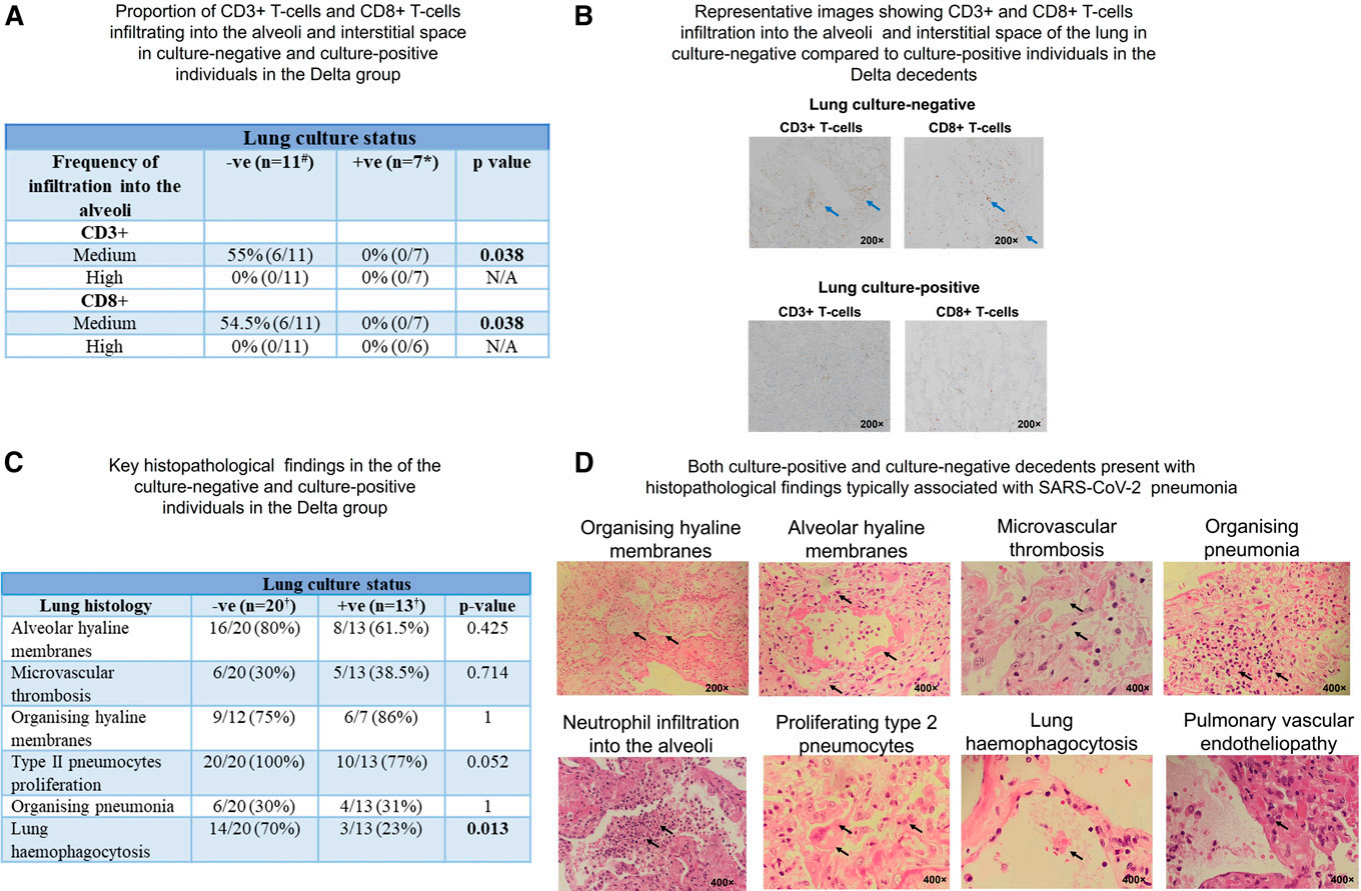
Higher proportions of T cells, macrophages, and pneumonocytes infiltrate into the lung of culture-negative versus culture-positive decedents in the Delta group. (*A*) More CD3^+^ (cluster of differentiation 3) and CD8^+^ T cells infiltrate into the alveoli and interstitial space of the lung culture–negative versus lung culture–positive group in the Delta decedents as assessed using immunohistochemistry (IHC). (*B*) Representative images (IHC) at 200× magnification showing increased T-cell infiltration into the interstitial space (blue arrow) in lung culture–negative versus lung culture–positive subjects in the Delta cohort. The density of CD3^+^ or CD8^+^ T cells in the alveoli or interstitial tissue were assessed and scored as medium or high. A magnitude of 10–50% or >50% was defined as medium or high infiltration, respectively. (*C* and *D*) Histopathology findings (*C*) and representative images (*D*) associated with diffuse alveolar damage and microvascular thrombosis in the Delta decedents. The arrows indicate key histopathological features. The magnification setting was either 200× or 400×. The relative magnification of each light microscopy image is shown. ^#^IHC was not performed on 5 culture-negative samples. *IHC was not performed on 1 culture-positive sample. ^†^Histology was not performed on all the biopsy samples. N/A = not applicable; SARS-CoV-2 = severe acute respiratory syndrome coronavirus 2; -ve = negative; +ve = positive.

We then ascertained whether the phenotype of replicating viral persistence was associated with attenuated or modulated lung immunity in the Delta decedents (transcriptomic and flow cytometric studies were performed only in Cape Town, i.e., the Delta decedents, because of location-specific availability of assays and limited Beta group biopsy cores that had been used for unrelated studies). Immunohistochemical staining indicated that there was significantly less infiltration of CD3^+^ T cells, specifically CD8^+^ T cells, in the alveoli and interstitium of the SARS-CoV-2 culture–positive compared with the culture-negative individuals in the Delta decedents (Figures 4A and 4B).

The typical histological features of severe COVID-19 (e.g., diffuse alveolar damage and microvascular thrombosis) were similar in the SARS-CoV-2 culture–positive and the culture-negative phenotypes, suggesting that these events occurred in the early rather than the persistent viral replication phase (Figures 4C, 4D, and E4 and Tables E2 and E3). Interestingly, we observed that some features of leukocyte hyperactivation (i.e., hemophagocytic syndrome) were more common in the SARS-CoV-2 culture–negative versus the culture-positive group, potentially in keeping with an aberrant immune response characterized by a lack of immune regulation, as outlined above (Figures 4C and 4D; *P* = 0.013).

The transcriptional analysis of postmortem lung tissue after adjustment for multiple testing identified a total of 11 up- and 4 downregulated genes in the culture-positive versus culture-negative groups (FDR < 0.05; specific genes are discussed further in the online supplement; *see* Figure E6). To ensure that the transcriptional signal was uniform, lung biopsy cores from each decedent were placed in one tube containing RNAlater (Thermo Fisher) to ensure that enough genetic material was obtained.

The lung culture–positive group expressed higher concentrations of CA12 (carbonic anhydrase 12) than the lung culture–negative group (*see* Figure E6 and Table E6). This protein induces a phenotype similar to high-altitude pulmonary edema, with a decreased ratio of Pa_O_2__ to Fi_O_2__, and a reduction of carbon dioxide concentration (29). This was associated with increased tachypnea and fibrinogen concentrations/fibrin formation and the presence of hypoxia leading to acute respiratory distress syndrome (ARDS) (29).

Another gene that was highly overexpressed in the culture-positive cohort was CD177, a glycosylphosphatidylinositol-anchored protein expressed by neutrophils. CD177 plays a key role in neutrophil activation, transmigration, and adhesion to the endothelium and is associated with the severity of COVID-19 (Figures 6 and E6; *see* Table E6) (30). Fu and colleagues (31) reported a high neutrophil-to-lymphocyte ratio in the alveolar spaces of the lung from deceased patients with COVID-19. Elevated concentrations of CD177 were recently identified by transcriptomics in the peripheral blood (32) and by proteomics in BAL cells (33) of patients with COVID-19 with mild and severe disease, which supports our data suggesting an upregulation of CD177 in the lung culture–positive decedents.

SDCBP2 (syndecan-binding protein 2) was significantly upregulated in the culture-positive versus the culture-negative group (*see* Figure E6 and Table E6). The protein is a family member of the syndecans (SDCs), which are transmembrane proteoglycans that facilitate the cellular entry of SARS-CoV-2 (34). Endothelial cells express SDC2 (syndecan 2) during virus internalization, and SDCs colocalize with ACE2 (angiotensin-converting enzyme 2), suggesting a jointly shared internalization pathway. Hudák and colleagues (34) reported that entry via SDCs enabled efficient gene transduction with SARS-CoV-2 pseudovirus, which implied that SDC-mediated internalization pathway maintained the viral particles’ biological activity. Viruses that target SDCs in the lung may therefore interfere with SDC-dependent signaling, as inhibitors of both ACE2 and SDC reduced the cellular entry of SARS-CoV-2, thus supporting the complex nature of internalization.

**Figure 5.**
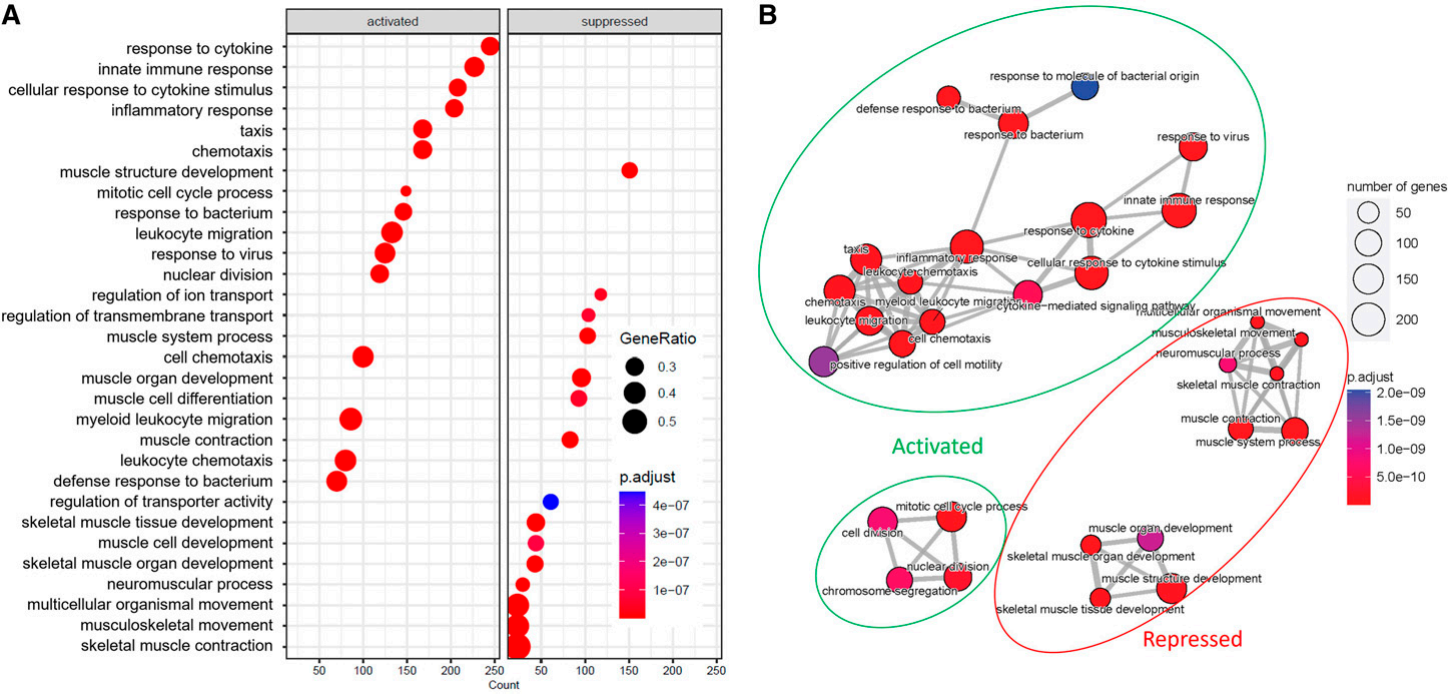
The transcriptomic analysis revealed that the culture-positive group, in comparison with the culture-negative group, had enrichment of activated pathways associated with inflammation, innate immunity, responses to cytokines, and responses to virus/bacterial stimuli in the Delta descendants. (*A*) Dot plot illustrating the significantly activated and suppressed pathways along with the gene count and ratio for each pathway. (*B*) Enrichment map illustrating the significantly activated and suppressed pathways together with the gene count and ratio for each pathway. SARS-CoV-2 = severe acute respiratory syndrome coronavirus 2.

**Figure 6.**
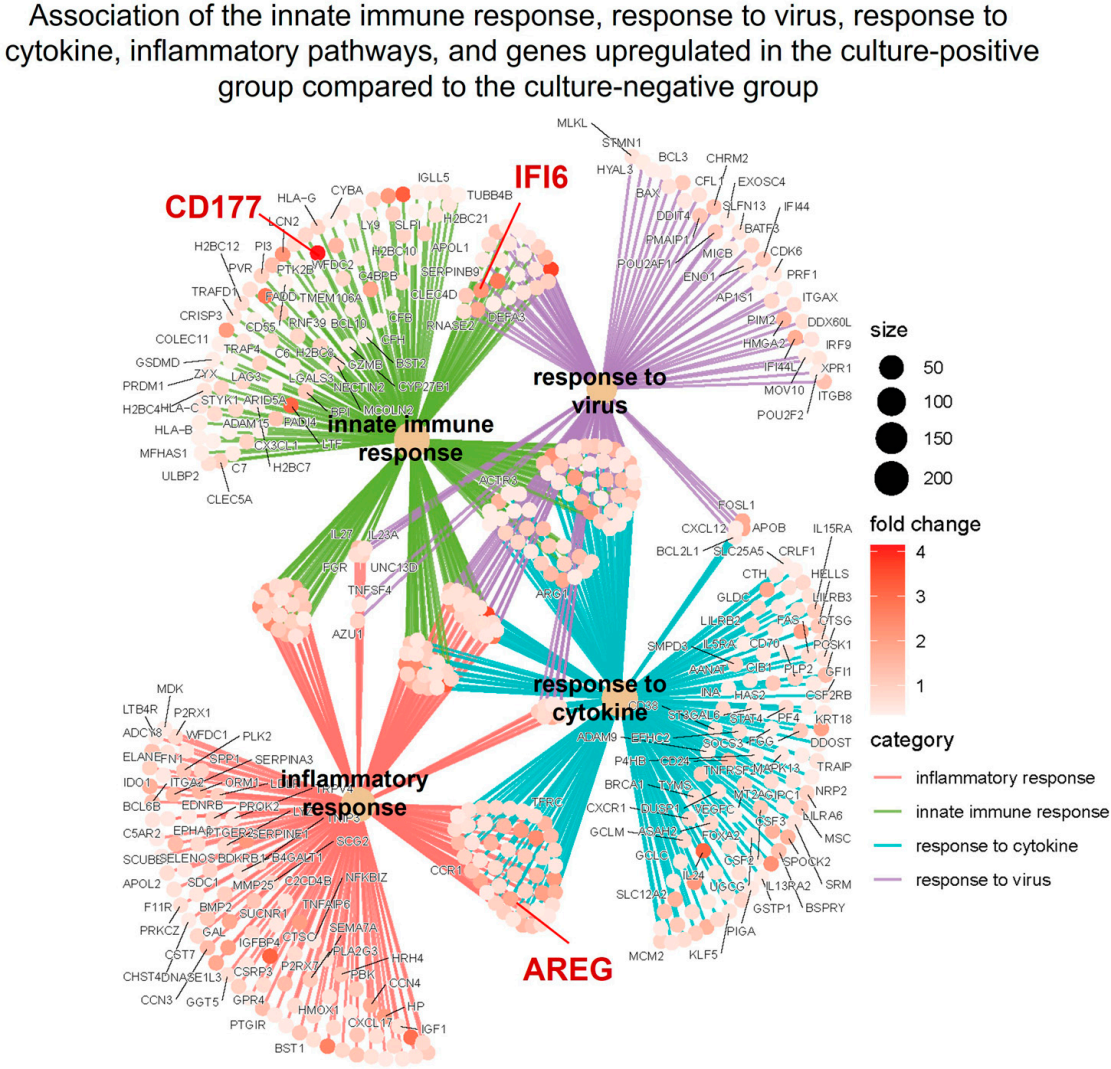
Transcriptomic analysis showing the association among the innate immune response, response to virus, response to cytokine, inflammatory pathways, and genes upregulated in the culture-positive group versus the culture-negative group. The cnetplot illustrates the overlap of genes and their fold changes for selected activated pathways. Significant genes (adjusted *P* < 0.05) that are annotated to the pathways are highlighted in red. CD = cluster of differentiation; LAG3 = lymphocyte activating 3.

The gene set enrichment analysis performed using the full list of differentially expressed genes ranked by fold change identified activated pathways that were associated with a proinflammatory response related to cytokine signaling, neutrophil and monocyte chemotaxis/recruitment, and viral entry/defense, all of which are implicated in COVID-19–related hypercytokinemia (35) (Figures 5, 6, E5, and E6; *see* Tables E6 and E7A). Significantly repressed pathways were generally associated with body homeostasis (Figures 5A and 5B). There was also in-tandem upregulation of T-helper cell type 1 and T-helper cell type 17 signaling pathways (*see* Table E7B) but to a substantially lesser extent than that of innate cellular and signaling pathways (IL-1, IL-6, and neutrophil related; *see* Table E7A). These features may be consistent with an aberrant immune response, including a lack of activation of regulatory and immune-suppressive pathways. T-cell exhaustion consistent with upregulation of PD-1 (programmed cell death 1), CTLA-4 (cytotoxic T-lymphocyte–associated protein 4), and LAG (lymphocyte activating) (*see* Table E7C), known to be associated with severe COVID-19, was not observed.

The DE results also revealed that a number of SARS-CoV-2 genes were significantly upregulated (FDR < 0.01) in the culture-positive versus the culture-negative group, including nucleocapsid phosphoprotein (log_2_ FC = 8.4) and *ORF3a* (log_2_ FC = 5.5), while the surface/spike glycoprotein encoding gene had a log_2_ FC of 5.3 and an FDR of 0.067 (*see* Table E6). A visual inspection of the mapped SARS-CoV-2 reads revealed that those that mapped to the 5′ end of the genes were spliced with a portion mapping to the 5′ leader sequence of the genome. This suggests that the reads originated from subgenomic mRNA (sgRNA) rather than genomic RNA, which is consistent with the active viral replication observed in the culture-positive group.

Finally, we evaluated whether any of the differentially expressed genes could act as biomarkers discriminating between lung culture–positive and lung culture–negative individuals. Logistic regression predictive modeling revealed that *GREM1* (gremlin 1, DAN family BMP antagonist) and *FGFBP1* (fibroblast growth factor–binding protein 1) were associated, with sensitivity and specificity greater than 90% (*see* Figure E6). Future studies are warranted to determine if these lung-based biomarkers can predict patient culture status in blood samples.

## Discussion

The widely accepted view in severe acute COVID-19 is that resolution of the initial viral replication phase in the first week after symptom onset is followed by an effector or hyperinflammatory phase in the second and third weeks of illness, which is characterized by diffuse alveolar damage, thromboinflammation, and endotheliopathy (36). Indeed, the Infectious Diseases Society of America recommends the use of remdesivir for only 5 days in patients with severe illness and not at all in mechanically ventilated patients (37). However, our results, based on postmortem lung biopsies obtained using minimally invasive tissue sampling methods shortly after death, indicated that in contradistinction to the URT, where replication often ceases within ∼8 days of symptom onset, in the human lung, virus is culturable in ∼40% of mechanically ventilated patients until death (a median of 15 days and up to 4 weeks after symptom onset; *see* Figure 1 for the study overview). To ensure reproducibility of the lung biopsy procedure, histological analysis was performed to confirm that the tissue was derived from the lung only (to ensure that there was no contamination from other tissue or muscle, which would have been detected on histopathological analysis at the least to some extent). The upregulation of muscle-associated gene pathways may have been related to virus-associated myositis or ICU-associated myopathy. The culture-positive group in the Delta cohort had accelerated death and a higher proportion of secondary bacterial infection in the lung compared with the culture-negative group. This may be explained by the Delta variant’s being more transmissible (38) and associated with enhanced replication, a higher viral load (39), and greater immune escape (40) than the Beta variant.

Nasopharyngeal SARS-CoV-2 viral load (on the basis of Ct value) at admission and at death was not predictive of lung culture positivity. SARS-CoV-2 culture positivity in the lungs of decedents was associated with attenuated pulmonary T-cell immunity and an exaggerated proinflammatory phenotype. Importantly, this was concurrent with, rather than sequential to, the viral replication or viral culture–positive phase.

These findings challenge the traditional paradigm of an initial viral replicative phase in the first week of severe illness sequentially followed by an effector or inflammatory phase (36). Our data suggest that in ∼40% of ventilated patients, viral replication persisted until death (i.e., the third and fourth weeks of illness and a median of 15 days after symptom onset) compared with ∼2 to ∼8 days in the URT as outlined in several studies, including a live-virus human challenge study (11, 14–24). One outlier study reported culturing virus from the URT for up to 3 weeks after symptom onset (41). However, a large proportion of patients were immunocompromised, samples at diagnosis and follow-up were combined (skewing the results), a high proportion of participants were healthcare workers (reinfection may have been a confounder), and, as the authors suggested, a limitation was that the Vero cell line used was overtly permissive to infection compared with the human lung carcinoma cell line H1299 ACE2, which is a biologically representative cell line (and one that we used). Another recent study showed that infectious virus production peaked in the human lung within 2 days, but this model used *ex vivo* agarose infused devascularized and explanted human lung slices, which are not representative of what is occurring in freshly harvested human lung (42). The culture-based findings in the aforementioned studies must be explicitly distinguished from studies that detected residual free viral genomic RNA (but not replicating virus) embedded in the respiratory tract tissue of patients who had severe disease for an extended period of time (13, 43, 44). Indeed, SARS-CoV-2 RNAs have been detected in patient tissue many months after recovery from acute infection (45–47). It was initially suggested that sgRNA (small strands of reverse transcribed RNA) could be used as a proxy to infer viral replication. However, several recent studies have indicated that it has poor predictive value as a proxy for viral replication (48, 49). Indeed, Stein and colleagues (13) detected sgRNA in multiple postmortem organ biopsies, including the brain, several months after symptom onset. Thus, the data presented in this paper shows for the first time, that we can culture virus conclusively and comprehensively using from lung tissue (the gold standard to detect viral replication) beyond two weeks after symptom onset.

We demonstrated active viral replication in the lungs of acutely ill ventilated patients for up to ∼4 weeks after symptom onset. This challenges the current practice of using antivirals such as remdesivir for only 5 days and suggests that a longer duration of treatment may be required in critically ill patients. Furthermore, antivirals such as remdesivir are not recommended for use by the Infectious Diseases Society of America in mechanically ventilated patients (conditional recommendation), as it was believed that such patients (often in the third week of their illness) are no longer in the viral replicative phase, and published controlled trial data showed no mortality benefit of remdesivir in such patients (37, 50). However, these studies demonstrated a group effect, and the analyses did not adjust for disease severity or the time from symptom onset to death in mechanically ventilated patients (51). Our data suggest that a significant number of patients will likely benefit from antivirals during mechanical ventilation. Indeed, several observational studies have demonstrated a survival benefit using remdesivir in mechanically ventilated patients, but this requires further clarification in appropriate trials (51–53). It is also possible that the very advanced immunopathology in some patients may render antivirals redundant. In a multivariate analysis, we found no association between steroid use and lung viral culture positivity; in fact, steroid use was lower in the viral culture–positive group (and there was a trend toward an inverse relationship in the multivariable analysis, and often culture positivity persisted beyond the 10 days of steroid use.

The transcriptomic data suggested that in a significant number of patients, the hyperinflammatory and viral replication phases occur concurrently in the third and fourth week of illness, in contradistinction to the widely held view that these are sequential phases. Antiviral and selective proinflammatory responses were overrepresented in the SARS-CoV-2 culture–positive compared with the culture-negative decedents, and we did not detect attenuated type 1 IFN responses at the site of disease, in contrast to other findings (54–58). Three prior studies (one that enrolled five patients with COVID-19) evaluated transcriptomic lung responses in patients with severe COVID-19 versus healthy control subjects (54, 55, 59). These first-level studies logically attempted to address the significance of transcriptomic changes specific to COVID-19 by using healthy control subjects or nondiseased parts of the lung from patients with lung cancer. However, we specifically sought to compare culture-positive versus culture-negative groups (hitherto not undertaken) to identify pathways that facilitate permissiveness to ongoing viral replication.

We identified two lung-based biomarkers (*GREM1* and *FGFBP1*) that could predict culture positivity. Although these are lung-specific biomarkers, this preliminary analysis in a limited number of samples suggests that in the future, RT-PCR of tracheal aspirates or blood (if they are concordant with lung findings), could potentially serve as biomarkers to identify and direct appropriate treatment protocols to culture-positive persons, but further investigation is needed.

There are several limitations to our findings. First, our findings are relevant to acute severe COVID-19 ARDS/pneumonia requiring mechanical ventilation and may not be applicable to milder forms of disease seen in hospitalized patients or chronic infection seen in immunocompromised patients.

Second, we studied only patients with the Beta and Delta variants, as these were the predominant variants at the time of the study. However, Omicron has also been associated with severe disease in several settings, including the surge of severe COVID-19 unfolding in China.

Third, we did not study a control group comprising severe ARDS due to other causes, because our express aim was to investigate the presence and duration of viral replication in the LRT in severe COVID-19. Fourth, the sample size limited our ability to make conclusions about several aspects. However, the highly resource intensive and demanding nature of the study limited our ability to recruit higher numbers of participants.

Fifth, it could be suggested that there may have been sampling error and variability of the viral culture assay. However, the reproducibility of the viral culture technique using six samples across two separate runs had a low SE, which was indicative of high reproducibility.

Sixth, we did not compare the culture status of the LRT in the ambulatory control subjects versus the decedents. This was due to ethical reasons and the potential risks of viral transmission to the medical and research staff during bronchoscopic procedures.

Finally, the transcriptional signature and flow cytometric findings may have been affected by postmortem sampling, but several detailed studies have shown (60) that most protein and RNA species are preserved and stable for several hours after death. Given that biopsies for the transcriptional studies were taken ∼2 hours after death, we believe that they are broadly representative of the picture at the time of death.

### Conclusions

In summary, our data suggest that in COVID-19, there is considerable heterogeneity in the frequency and duration of viral replication in the URT versus the LRT (i.e., lungs) beyond the second week of illness and that in a significant proportion of seriously ill patients, persisting viral replication occurs concurrently and may drive an exaggerated proinflammatory response (higher than in culture-negative persons), rather than sequentially, as is widely believed. These findings have potential implications for the use of antiviral therapy in seriously ill patients with COVID-19 and suggest that better biomarkers are needed to identify patient phenotypes and subsets that might benefit from concurrent antiinflammatory and antiviral therapy.
